# Ovarian cancer: diagnostic accuracy and tumor types distribution in East Africa compared to North America

**DOI:** 10.1186/s13000-020-01000-3

**Published:** 2020-07-16

**Authors:** Peter F. Rambau, Martin Köbel, Derek Tilley, Alex Mremi, Robert Lukande, William Muller

**Affiliations:** 1 Department of Pathology, Catholic University of Health and Allied Sciences (CUHAS), Box 1464, Mwanza, Tanzania; 2 Department of Pathology and Laboratory Medicine, Foothill Medical Center, University of Calgary, 1403 - 29 Street NW, Calgary, AB Canada; 3 Holy Cross Cancer Center, 2210 2nd St. SW, Calgary, AB Canada; 4 Department of Pathology, Kilimanjaro Christian Medical Center, Box 3010, Moshi, Tanzania; 5 Department of Pathology, College of Health Sciences Makerere University, Box. 7072, Kampala, Uganda; 6 Department of Pathology, Mbeya Referral Hospital, Box 419, Mbeya, Tanzania

**Keywords:** Ovarian cancer, Histotypes, Immunohistochemistry, Diagnostic accuracy

## Abstract

**Background:**

Ovarian cancer is a spectrum of several histologically distinct tumor types that differ in etiology, response to therapy, and prognosis. In resource-limited settings, the diagnosis of ovarian cancer can be challenging. This study describes the distribution of ovarian cancer tumor types in East Africa as well as assessing the diagnostic accuracy by using contemporary methods.

**Methods:**

Data from 210 women identified from the records with a diagnosis of ovarian cancer in a period of 15 years were included. Two tissue microarrays were constructed and stained with 20 antibodies relevant to ovarian cancer subtyping. An integrated diagnosis was reached by the review of full Haematoxylin and Eosin stained sections, with consideration of immunohistochemical results. The integrated diagnoses were compared with the original diagnoses, and the degree of agreement was evaluated by percentage and Kappa statistics.

**Results:**

Though limited by selection bias, the results suggest lower rates of ovarian cancer in East Africa compared to a North American population from Alberta, Canada. There was a higher proportion of sex cord stromal tumors and germ cell tumors in the East African population. Diagnostic accuracy for main ovarian tumor type categories was substantial (Kappa 0.70), but only fair for specific ovarian carcinoma histotypes (Kappa 0.34). Poor Haematoxylin and Eosin stain was the main factor hindering the correct diagnosis, which was not related to tissue processing.

**Conclusions:**

In a resource-limited setting, where immunohistochemistry is not routinely carried out, diagnostic accuracy for the main categories of ovarian carcinoma is substantial and could be further improved by standardization of the basic Haematoxylin and Eosin stain.

## Background

There is considerable regional variation in the incidences of ovarian cancer, whereby the highest rates are seen in Europe (10.1 per 100,000) and North America (8.7 per 100,000). In contrast, the lowest incidences are seen in Japan and developing countries [1]. Data for Africa are limited as there are fewer cancer registries and cancer control programmes. In Africa, cancer statistics are based mainly on estimates [2, 3], which show a lower rate of 4.2 per 100,000. In Sub-Saharan Africa, the burden and types of ovarian cancer are largely unknown. In the East African countries, data from Kenya shows that ovarian cancer constitutes 3.4% of all cancers. However, these data are based on cancer registries located in major urban cities and do not include the rural population [4].

The global distribution of ovarian cancer tumor types has been documented, and the majority of ovarian malignancies are epithelial [5]. Ovarian cancers are further subtyped into histotypes, which proved to be predictors for treatment choice, prognostication, and genetic counselling. Correct diagnosis and typing are, therefore, becoming mandatory [6]. Historically, there has been misclassification of ovarian carcinoma [6], with the African data considered unreliable, and the influence of local diagnostic practice is unknown. The cancer burden in African countries is increasing [7], and health facilities are facing significant challenges in many Sub-Saharan African countries [3]. Pathology services in Africa show significant gaps of qualified professional and technical staff, inadequate infrastructure, and low operational funding [8–10]. Cancer treatment rests on an accurate diagnosis, which represents a major challenge for the increasing number of cancer patients.

This study aimed to assess the frequency and diagnostic accuracy of understudied ovarian cancer types in selected centers in Tanzania and Uganda, compared to a population from Alberta, Canada.

## Methods

### Study sites

This retrospective study involved cases of ovarian cancer diagnosed in the year 2002 to 2017 from three centers in Tanzania: Bugando Medical Centre (BMC), Kilimanjaro Christian Medical Center (KCMC), and Mbeya Referral Hospital, and the Pathology Department at Makerere University, Kampala, Uganda. These are the only tertiary hospitals with diagnostic facility for cancer, all patients or samples of patients with cancer are brought to these centers for diagnosis. Bugando Medical Centre serves the North-Western area of Tanzania with a population of 13 million; KCMC serves a population of 11 million from the North-Eastern part of Tanzania; and the Mbeya Referral Hospital serves a population of 8 million from the Southern Highlands of Tanzania. In Uganda, the Pathology Department of Mulago Hospital serves a population of 3 million.

### Pathological and clinical information

A search was undertaken for cases of ovarian cancer diagnosed from 2002 to 2017 from the files of the pathology department at each participating center. For each ovarian cancer diagnosis identified, the histological type was recorded as well as the demographic characteristics of the patient. This process was completed by searching the medical records from the patient files. By using the histology number, a corresponding formalin fixed paraffin embedded (FFPE) tissue block and haematoxylin and eosin (H&E) stained histological slides were retrieved from the archival pathology materials. In circumstances where the H&E stained slides were not available, new sections were made and stained by H&E. All H&E stained slides were examined under a light microscope, and the slides with a viable representative tumor were selected and matched with the corresponding FFPE tissue block. In each case, one to three H&E stained slides and FFPE tissue blocks were selected for the study. The selected FFPE tissue blocks and corresponding H&E stained slides were shipped to the Anatomic Pathology Research Laboratory (APRL) of the University of Calgary, Alberta, for further studies.

### Tissue microarray (TMA) construction

In each case, one good representative H&E stained slide was selected and marked for an area with high tumor cellularity. The corresponding area on the FFPE tissue block was selected and punched with a 0.6 mm needle core for TMA construction. Two tissue microarrays containing 0.6 mm duplicate cores from each case were constructed. This procedure was carried out at the Anatomical Pathology Research Laboratory (APRL) of the University of Calgary by a Semi-automated Tissue Arrayer (TMArrayer™, Pathology Devices, Inc. USA).

### Immunohistochemistry (IHC)

Antigen retrieval, staining platforms, antibody dilutions, detection methods, and interpretation of the staining are shown in Supplementary Table S-1. In brief, 0.4-μm sections were made from the two TMA’s, and staining was carried out by the Dako Omnis Platform of the Calgary Laboratory Services, and the APRL of the University of Calgary. The TMA’s were stained with the following antibodies: antibodies for general interest in ovarian cancer AE1/AE3 and PAX8, and antibodies specific for ovarian carcinoma typing as described previously [11]: WT-1, p53, Napsin A, PR, p16, TFF3, ARID1A, and Vimentin. More antibodies to complement typing for ovarian carcinoma were added: ER, Mismatch repair proteins (MSH6 and PMS2) and SATB2. For germ cell tumors and Sex-cord Stromal tumors, the following antibodies were included: Inhibin, FoxL2, Melan A, OCT-4, and GLYP3. CD45 and c-myc were used for lymphomas as well as BRG1 for small cell carcinoma hypercalcemic type.

### Histotype assignment

Full section H&E stained slides from each ovarian cancer cases were reviewed under light microscopy by the investigators. The morphology of ovarian cancer was assessed based on the 2014 WHO classification of tumors of the female reproductive tract and complemented by immunohistochemical expression patterns as previously described [11]. In a situation where there was discordance between morphology and immunophenotype, a consensus was reached by the arbitration of a third reviewer.

### Alberta cancer registry

We identified all patients diagnosed with ovarian cancer in Alberta between 2008 and 2016 from the Alberta Cancer Registry. Patient demographics and treatment data were extracted from the Discharge Abstract Database, the National Ambulatory Care Reporting System Database, or were obtained directly from electronic medical records (ARIA RO/MO).

### Statistical analysis

Data was entered and cleaned using a Microsoft Excel spreadsheet, and the analysis was performed with statistical software STATA 13 (College Station, TX, USA) and JMP12 (SAS, Cary, NC, USA). For analysis, categorical variables were summarized as the proportions, and continuous variables were summarized as means with standard error. The statistical tests were performed by two-sided Chi-square test for categorical data and two-sided student-t tests for the continuous data. The statistically significant differences were considered when *p*-values were less than 0.05. Agreement between original diagnosis and the revised diagnosis was assessed as a percentage of cases agreed upon (concordance), and by Cohen’s kappa.

## Results

### Study composition and patients’ characteristics

**Fig. 1:**
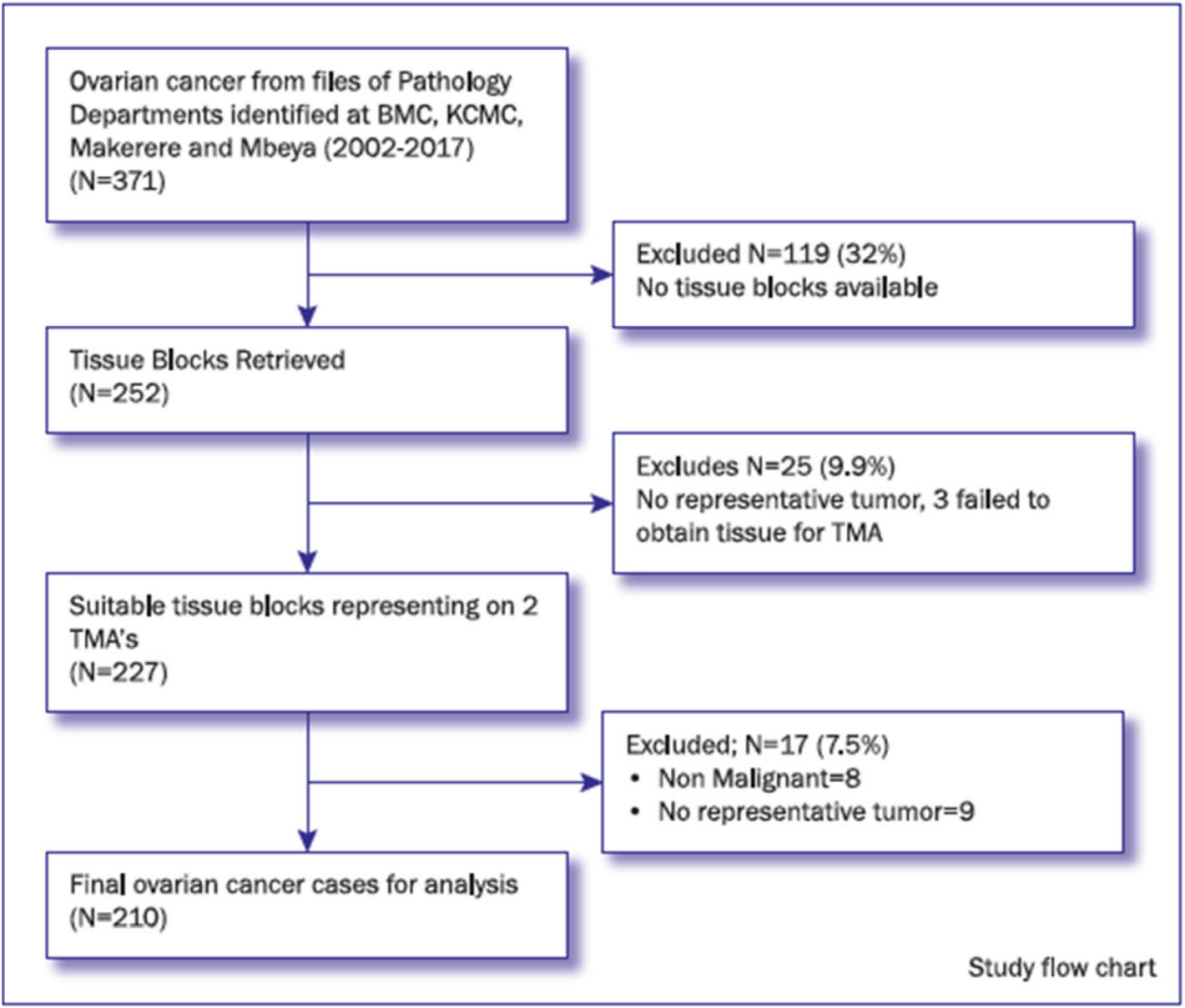
Study flow chart

A total number of 371 patients with a diagnosis of ovarian cancer made between 2002 and 2017 were identified from the study sites. Following exclusions, two TMA’s were constructed representing 227 ovarian cancer cases. Further exclusions resulted in 210 ovarian cancer cases suitable for analysis, as shown in Fig. 1. For all patients enrolled, the median age was 47.5 (Range 6 to 86) years. The patients presented with various symptoms and the most common symptoms were abdominal swelling, abdominal pain, abdominal distention, and abnormal uterine bleeding. The duration of the condition ranged from 2 weeks to 84 months, with a median duration of 6.5 months.

Menopausal status was available for 91 patients only, whereas 34 (37.4%) were post-menopausal. All patients underwent surgery, and the majority of the specimens received for investigation were ovarian masses 126 (60%), followed by hysterectomy and bilateral salpingo-oophorectomy. In a few cases, staging surgery was accompanied by omentectomy, bowel resection, and lymph node sampling. The stage of the disease was available in four patients only, and all were at an advanced stage. There was no data on disease recurrence or survival.

### Ovarian tumor types in original and revised diagnoses

In the original diagnosis, the majority of ovarian cancers were epithelia (carcinomas), which constituted 147 (70%) of all the cases with a mean age of 50.5 (SE ±1.2) years. The second most common diagnosis was a sex cord stromal tumor in 24 (11.4%) cases with a mean age of 37 (SE ± 3.0) years, followed by germ cell tumors 21 (10%), with a mean age of 22.8 (SE ± 3.2) years. The diagnosis of ovarian lymphomas was rendered in 14 (6.7%) of the cases with a mean age of 15.9 (SE ±3.7) years. Other types included sarcoma in 2 (0.9%) cases and unspecified cancers in 2 (0.9%) cases.

Following morphological review and IHC integration, there was substantial agreement between original diagnosis and a revised diagnosis in the major categories of ovarian cancer (Kappa 0.702 with 95% CI of 0.619 to 0.791), as shown in Table 1. In a revised diagnosis, the largest category was still epithelia in 127 (60.5%) of all the cases with a mean age of 52 (SE ±1.2) years. The second most common category was sex cord stromal tumors with 30 (14.3%) cases with a mean age of 45 (SE ±2.9) years, followed by germ cell tumors 27 (12.6%), and lymphomas 13 (6.2%). There was no significant mean age difference from the original to the revised diagnosis between ovarian tumor types.

**Table 1:** Original diagnosis and IHC integrated revised ovarian cancer types

	Revised diagnosis									
	EPH	GCT	LYMH	SCST	SC	Others	NM	Total	Concordance (%)	Kappa
**Original Diagnosis**										
**EPH**	123	9	0	9	0	0	6	147	83.7	
**GCT**	3	17	0	1	0	0	0	21	81.0	
**LMPH**	0	0	13	0	1	0	0	14	92.9	0.7019
**SCST**	1	1	0	20	1	0	1	24	83.3	
**SC**	0	0	0	0	2	0	0	2	100.0	
**Others**	0	0	0	0	1	1	0	2	50.0	
**Total**	127	27	13	30	5	1	7	210		
**Concordance (%)**	96.6	63.0	100	66.7	40.0	100.0	NA			

*EPH* Epithelia, *GCT* Germ Cell Tumors, *LMPH* Lymphomas, *SCST* Sex Cord Stromal Tumors, *SC* Sarcomas, *NM* Non Malignant

### Histotype specific agreement between original diagnosis and revised diagnosis

**Fig. 2:**
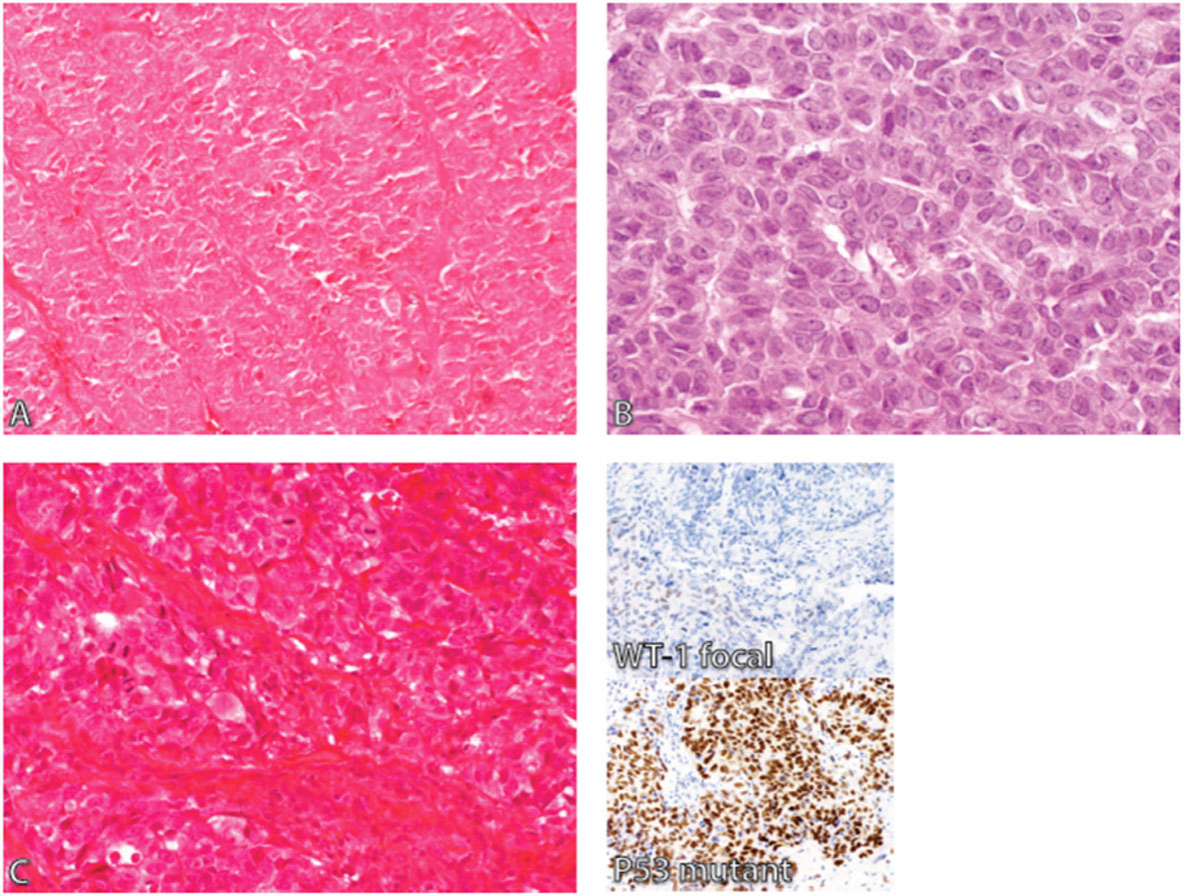
The case (**a**) shows a poor morphology diagnosed as carcinoma NOS, and following re-stain (**b**), the morphology was that of adult granulosa cell tumor. **c** is another case that shows poor morphology with obvious mitotic figures diagnosed as carcinoma NOS, and IHC shows focal WT-1 stain and p53 mutant type staining pattern

**Fig. 3:**
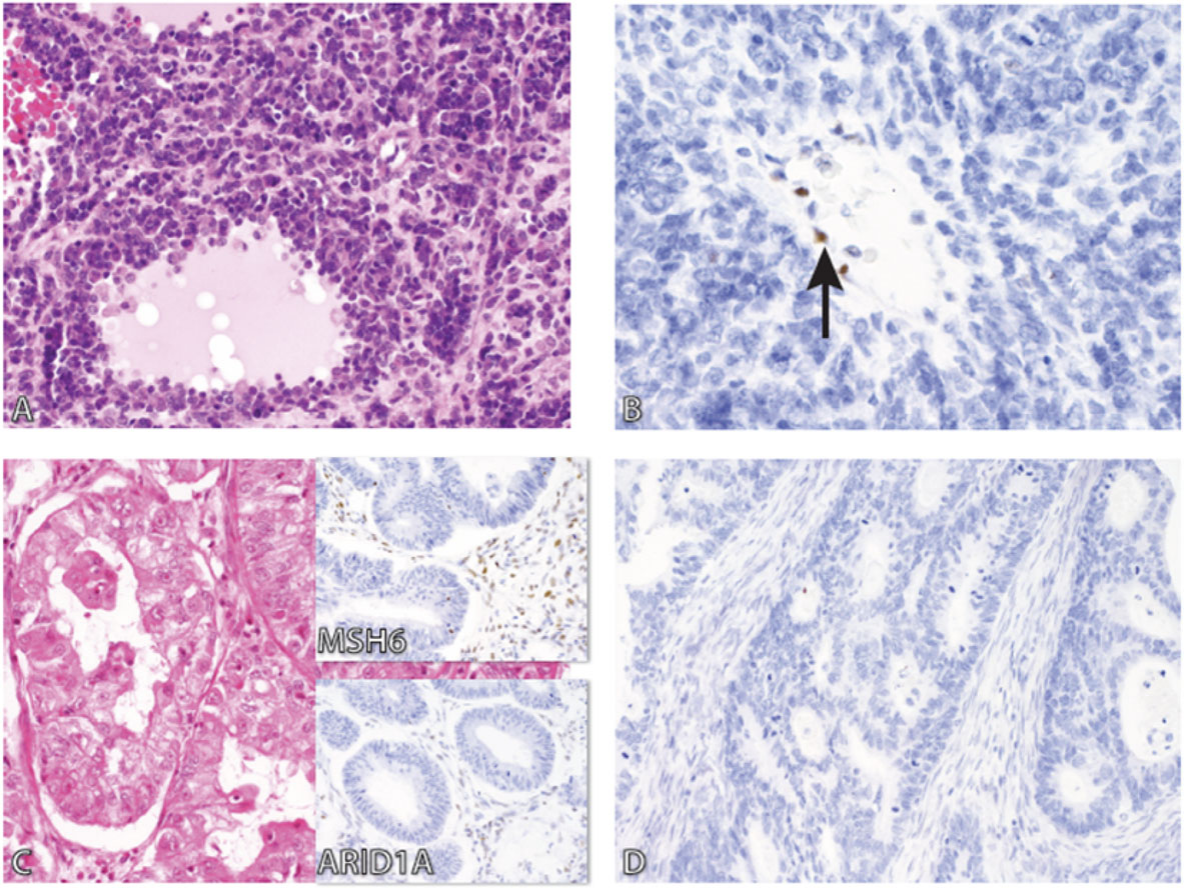
A case of SCCOHT showing diffuse small cells with dark nuclei and pseudo follicles with eosinophilic material (**a**), and the loss of BRG1 staining in tumor cells (**b**) and positive staining of stroma cells (Arrow). **c** shows a case on EC with mucinous features (initially diagnosed as MC) with loss of MSH6 and ARID1A (insets) in tumor cells with positive staining in stromal tissue and lymphocytes. D is a case with Loss of ARID1A with no internal control (no staining of stromal tissue or lymphocytes)

In a revised diagnosis, the specific histotypes were assigned based on 2014 WHO Classification of Tumors of Female Reproductive Organs. There was a fair agreement between original diagnoses and revised diagnoses (Kappa = 0.343, 95% CI: 0.277 to 0.409), as shown in Supplementary Table 2. In summary, a good concordance was seen in Lymphomas, followed by Germ Cell Tumors (92.9 and 81%, respectively). For epithelial tumors (carcinoma), a total number of 84 (57.9%) cases were not classified (carcinoma not otherwise specified (NOS)). Following review, a total number of 36 (42.9%) cases were reclassified to High Grade Serous Carcinoma (HGSC). Significant numbers of carcinoma NOS had poor H&E stains, in which the diagnosis was straightforward following recut and new H&E stain at Calgary laboratory. Two cases had a diagnosis of carcinoma NOS. Following H&E re-stain, one was adult granulosa cell tumor, and the other was HGSC with positive staining with WT-1 and p53 mutant type (see Fig. 2). There were 13 cases of HGSC in the original diagnosis, and the concordance with the revised diagnosis was 76.9%. Among 17 cases originally diagnosed as Endometrioid Carcinoma (EC), the concordance with a revised diagnosis was 58.8%, and 5 (29.4%) were reclassified to HGSC. The concordance of 21.7% was seen in Mucinous Carcinoma (MC), and 6 (26.1%) were reclassified to EC, whereas 4 (17.4%) were metastasis from GI. There were three cases of Low Grade Serous Carcinoma (LGSC), with a concordance of 33.3%, one case was EC, and the other was a metastasis from GI. There was one case of neuroblastoma which was reclassified as small cell carcinoma hypercalcemic type (SCCOHT) with loss of BRG1 staining in tumor cells and positive staining of stromal cells (See Fig. 3a & b), and one case of carcinoma NOS was reclassified as mixed carcinoma (EC/LGSC).

### IHC markers expression in ovarian cancers

The patterns of IHC markers expression across different ovarian cancer histotypes are shown in Table 2. PAX8 was expressed in all LGSC, in 87% of HGSC and in none of the mucinous carcinomas. The Fallopian tube marker WT-1 was positive in 89% of HGSC, all LGSC, and in 27% of EC, with no expression in clear cell carcinoma (CCC) and MC. Hormonal receptor expression, estrogen receptor (ER) was higher (75%) in HGSC compared to other histotypes. For endometrioid carcinoma, ER and progesterone (PR) receptors were observed in 34 and 21% of cases, respectively, and one case of EC with mucinous features had a loss of MSH6 and ARID1A in tumor cells with a positive stain in stromal tissue and lymphocytes (See Fig. 3c). Abnormal staining of p53 was observed in 63% of HGSC, 18% of the EC, 20% of MC, and none of the LGSC. In sex cord stromal tumors, FOXL2 was expressed in 66 and 50% of adult granulosa cell tumor (AGST) and juvenile granulosa cell tumor (JGCT), respectively. SATB2 expression was predictive of ovarian metastatic mucinous tumor (*p* < 0.0001). Additionally, two cases of immature teratoma and one case of AGCT were positive for SATB2.

**Table 2:** Immunohistochemical marker expression across histotypes

Marker	Carcinoma (%)					Sex Cord Stromal Tumors (%)	
	HGSC	EC	CCC	MC	LGSC	AGCT	JGCT
PAX8: Positive	87.2	28.1	50	0	100	8.3	25
WT-1: Positive	89.5	27.6	0	0	100	47.8	0
p53: Mutant	63.2	18.8	0	20	0	4.2	0
p16: Abnormal	75.4	53.1	50	60	50	54.2	75
Napsin-A: Positive	0	3.1	25	0	0	0	0
ER: Positive	75.4	34.4	25	10	50	37.5	50
PR: Positive	19.3	21.9	0	0	0	33.3	0
ARID1A: Negative	0	3.1	0	0	0	0	0
Vimentin: Diffuse	14	21.9	25	0	50	95.8	75
MSH6: Negative	0	3.1	0	0	0	0	0
PMS2: Negative	0	0	0	0	0	0	0
FOXL2: Positive	0	0	**0**	10	0	66.7	50

For analysis of antibody performance, the known positive controls from APRL containing tonsil, Fallopian tube, placenta, and endometrium were included in each TMA. The performance of tested samples was assessed based on presence of internal controls for the tests, which require interpretation with internal controls (p53, ARID1A, MSH6, PMS2, and BRG1). All markers were not assessed equally across all the samples, as some samples were uninterpretable, or there was a loss of cores in the TMA. However, the majority of the samples were assessed for these markers, as shown in Table 3.

**Table 3:** IHC markers with no internal positive controls and level of the hospital

		Hospital level		
Marker	N (%)	Non-Tertiary (***N*** = 72)	Tertiary (***N*** = 147)	***p***-value
p53 (*N* = 204)	18 (8.8)	3	15	0.178
ARID1A (*N* = 208)	99 (47.6)	50	49	0.000**
MSH6 (*N* = 208)	15(7.2)	6	9	0.353
PMS2 (*N* = 206)	26 (12.6)	9	17	0.913
BRG1 (*N* = 201)	30 (14.9)	12	18	0.716

**Majority of cases with positive internal control were from tertiary hospitals (87/109)

The most affected marker was ARID1A, whereby 47% of the cases had a loss of staining in tumor cells as well as stromal tissue and lymphocytes, which are the internal controls (See Fig. 3d), followed by PMS2 (12%), and BRG1 (14%). When we analyzed for the primary site of sample fixation in relation to loss of internal control, there was no significant difference when the samples were fixed in tertiary or non-tertiary hospitals. However, for ARID1A, most of the samples with positive controls had primary fixation in a tertiary hospital. These results are summarized in Table 3.

### Comparison of ovarian tumor types between east African and Alberta

The comparison of ovarian cancer tumor types and histotypes distribution was performed using the following two populations: i) accessible ovarian cancer cases from East Africa diagnosed over a period of 15 years, and ii) Alberta Cancer Registry for cases diagnosed over a period of 9 years (2007 to 2016). In Alberta, the women population at risk of ovarian cancer is estimated to be 1.4 million women compared to the catchment area of the selected centers in East Africa of 9 million women at risk. There were 8-times more ovarian cancer cases in Alberta, compared to the selected population in East Africa, which is 6-times larger than the population of Alberta. This rough estimate suggests that there are 48 times more ovarian cancer cases in the Alberta population compared to the selected East African population. The distribution of the major tumor types in both populations showed epithelial tumors being the commonest, 89.9% in Alberta, and 58% in East Africa.

For the carcinomas, when a comparison was made by specific histotypes, HGSC occurred with the same frequency in both populations, as shown in Table 4. Still, EC was more common in the East Africa population than the Alberta population (28.3% versus 11.3% respectively).

**Table 4:** Patterns of ovarian cancer tumor types in Tanzania compared with Alberta, Canada

	Major categories			
	East Africa (n %)	Mean age (±SE)	Alberta (n %)	Mean age (±SE)
Epithelial	127 (60.5)	52.0 (1.2)	1645 (89.9)	61.8 (0.4)
Germ cell tumor	27 (12.6)	24.2 (2.7)	58 (3.2)	31.2 (1.5)
Lymphoma	13 (6.2)	15.3 (3.7)	NA	NA
Sex cord stromal tumor	30 (14.3)	45.0 (2.9)	17 (0.92)	57.1 (4.1)
Sarcoma	5 (2.4)	50.3 (6.7)	5 (0.3)	62.2 (6.1)
Others	1 (0.5)	30.0 (13.4)	104 (5.7)	79.0(1.2)
**Histotypes specific**				
**Carcinoma (epithelia)**	***N*** **= 113**		***N*** **= 1646**	
HGSC	57 (50.4)		831 (50.5)	
EC	32 (28.3)		186 (11.3)	
CCC	4 (3.5)		132 (8.0)	
LGSC	2 (1.8)		15 (0.9)	
MC	10 (8.8)		120 (7.3)	
Others	8 (7.1)		362 (22.0)	
**Germ cell tumors**	***N*** **= 26**		***N*** **= 58**	
Dysgerminoma	5 (19.2)		17 (29.3)	
Yolk Sac Tumor	14 (53.8)		10 (17.2)	
Immature Teratoma	4 (15.4)		18 (31.0)	
Others	3 (11.6)		13 (22.4)	
**Sex cord stromal tumor**	***N*** **= 30**		***N*** **= 17**	
Adult granulosa Cell Tumor	24 (80.0)		12 (70.6)	
Sertoli Leydig Cell Tumor	2 (6.7)		4 (23.5)	
Juvenile Granulosa Cell Tumor	4 (13.3)		0	
Others	0		1 (5.9)	

There was no striking difference for CCC, LGSC, and MC, and these cases occurred at a lower frequency in both populations. For Germ Cell Tumors, there was a predominance of Dysgerminoma and Immature Teratoma in the Alberta population (29.3 and 31%, respectively), compared to the East Africa population (19.2 and 15.4% respectively). However, the Yolk Sac Tumors were more common in East Africa compared to Alberta (53.8 and 17.2%, respectively). The Adult Granulosa Cell Tumor was the commonest sex cord stromal tumor in both East Africa and Alberta populations (80 and 70.6%, respectively), though in relatively low numbers in Alberta compared to the numbers of ovarian cancer cases. Juvenile Granulosa Cell tumors were exclusively seen in East Africa in 13.3% of the cases of SCST.

## Discussion

This study shows extremely few cases of ovarian cancer in East Africa compared to the Alberta population. There were limitations, and likely but unknown bias influencing the results. The age-standardized incidence rate could not be calculated for the East African population because of the lack of census data. On the one hand, a large proportion of the female population in East Africa may not yet be at the age of risk for ovarian cancer. On the other hand, the proportion of older females who carry the highest risk is likely to be lower in East Africa compared to Alberta [12]. The WHO 2014 cancer country profiles show that the life expectancy of Canadian women is 20 years higher compared to East African women. The larger population per cancer center in East Africa and other impediments such as access to health care facilities will most likely result in under-diagnosis of cases. For example, older women in rural areas may die of undiagnosed disease, unaware of the significance of the symptoms, and lacking the resources to seek medical attention [9]. While our data provide the first benchmark of ovarian cancer in East Africa, these limitations lead us to assume that cases from selected areas in East Africa may not be representative of the whole population. Therefore, there is a need to establish cancer registries and census data in Eastern Africa, similar to those in developed countries.

Ovarian cancer shows a wide geographical variation with a higher incidence in North America compared to Asia and Africa [13]. In this study, the Alberta population had more cases of epithelial cancer (89%) compared to East Africa (60%). In contrast, germ cell tumors and sex cord stromal tumors were more common in East Africa compared to the Alberta population. A large population-based study (CONCORD-2) reported a high proportion of germ cell tumors in Asia (4.2%), compared to Europe (1.3%) and North America (2.0%). In this study, we report a high proportion of germ cell tumors in East Africa (12.5%), similar to the one reported in Russia (11.4%) [14]. Similarly, the proportion of sex cord stromal tumors was 14.3% for East Africa, and 0.92% for the Alberta population. Because germ cell and sex cord stromal tumors occur at a younger age, this could reflect the age distribution of the underlying population and selection bias. There is a possibility that younger women with germ cell tumor or sex cord stromal tumor might seek medical attention and receive treatment, while older women with advanced epithelial cancer may not.

Morphology-based diagnosis of cancer remains a mainstay in developing countries, and ancillary immunohistochemical tests are rarely available. The diagnostic accuracy of ovarian cancer in East Africa showed a substantial agreement with the diagnosis of major categories of ovarian cancer but was only fair for specific histotypes. This agreement is a remarkable fact, given the inadequate laboratory resources in these settings, compared to North America, exemplified by the tremendous differences in the quality of the basic H&E stains. The fact that pathologists in East Africa can make a highly accurate diagnosis with virtually zero contrast H&E stains is an example of the remarkable adaption and ingenuity of the human brain. There is no reason to believe that they could not achieve a similar diagnostic accuracy regarding more specific histotyping with simple standardization of the H&E staining protocol.

In the past 15 years, there has been a significant change in the understanding and diagnosis of ovarian cancer, such that histotype has emerged as an important prognostic and predictive marker [15, 16]. Historically, the reproducibility of diagnosis of ovarian carcinoma based on the cell types [17–19] was low, but nowadays, the inter-observer reproducibility is excellent, and if not, it can be improved by the use of ancillary immunohistochemistry tests [20]. In this study, the morphological diagnosis of ovarian carcinoma was only fair, likely a result of a failure to use the current criteria for tumor cell types in routine practice [21], and the majority of the cases of carcinoma were unspecified. Significant numbers of unspecified carcinomas (~ 43%) in this study were reclassified as HGSC in a revised diagnosis, and these are the diagnoses likely made before 2014, where serous carcinomas were separated into HGSC and LGSC. For instance, a study performed in Canada necessitated a review of diagnoses; the majority (78%) of unspecified carcinomas based on 2003 WHO classification were reclassified to HGSC [22]. The problematic areas in ovarian carcinoma histotyping remain the differentiation of HGSC versus high grade EC and vice versa [6, 20, 23]. In the current study, 29% of the EC were reclassified to HGSC, contrary to 17% in a study carried out in Canada [22].

Mucinous carcinoma showed a poor concordance between the original diagnosis and the revised diagnosis, and a significant number (26%) were reclassified to EC, whereas 17% were metastatic gastrointestinal neoplasms. Differentiating primary ovarian MC from metastatic adenocarcinomas has been an area of challenge. In previous years, the diagnosis of MC was made frequently, constituting up to 14% of ovarian carcinoma [24]. Currently, with an improved understanding of ovarian carcinoma histotypes, and the use of ancillary tests, MC has become a rare subset of ovarian carcinoma, making it less than 5% of the cases [25–27]. In this study, we used SATB2, a recently identified marker, for differentiating primary ovarian from secondary colorectal/appendiceal tumors [28, 29]. The tumors which showed mucinous, or endometrioid like morphology suspicious for metastasis with an expression of SATB2 were considered as metastatic.

In this study, the cases of MC, which were reclassified as EC, were probably related to a failure of recognition of confirmatory endometrioid features or overreliance on features like mucinous differentiation, which do occur in EC [30, 31]. Furthermore, mucinous carcinoma with unapparent intracytoplasmic mucin can also mimic EC [27], and two cases which were initially diagnosed as CCC were actually endometrioid carcinoma with clear cell changes [30].

For non-epithelia tumors, the reproducibility in assigning tumor types was substantial, with the highest concordance in lymphomas (92%), followed by sex cord stromal tumors (83%), and germ cell tumors 81%. We did not go into specific histological types in these smaller categories because of lower case numbers but noted a high number of primary ovarian lymphomas.

This study identified one case of ovarian small cell carcinoma hypercalcemic type (SCCOHT), which was originally diagnosed as neuroblastoma. This lethal cancer, which affects young women, is characterized by an aggressive clinical course, poor prognosis, and a recently discovered pathognomonic mutation in *SMARCA4* [32–34]. The reasons for such diagnosis could be the morphology of a round dark cell tumor, which can easily attract a differential diagnosis of neuroblastoma in a setting where IHC tests are not routinely performed. Yet, ovarian neuroblastoma at the age of 30 years is quite uncommon, although it has been reported as primary ovarian or associated with mature teratoma in a few cases [35, 36]. Lack of awareness for the existence of this diagnosis in the current classification was a likely contributing factor, as well as the rarity of this tumor, noting that most of the pathologists would see at most one such case during their practice [21]. Foremost, this case illustrates the inability to render a correct diagnosis without access to confirmatory molecular testing for molecularly defined rare cancers.

This study applied various IHC markers used in ovarian cancer diagnosis and histotyping. The expression patterns followed the expected trend with some differences. At current, *TP53* mutations are ubiquitously present in HGSC [37, 38], and optimized p53 immunohistochemistry is used as a surrogate marker of *TP53* mutation [38, 39]. A notable difference was seen in the frequency of mutant p53 staining; only 63% of HGSC had mutant p53, whereas, in EC, it was seen in 18% of the cases as expected [22]. The low rate of abnormal p53 seen in HGSC could be due to various reasons. Some cases lost internal control, which was even commonly observed with ARID1A. In this study, we used a p53 optimized antibody, and a standardized staining platform, as well as known positive and negative controls. Therefore, poor staining and loss of internal control cannot be explained by the analytical process; the issue probably occurred in pre-analytical phases specifically related to tissue fixation. In East Africa, there is no uniform, standardized protocol for tissue fixation. The samples are sent from various hospitals to the pathology centres, and the quality of formalin and time for fixation vary considerably. Furthermore, FFPE tissue blocks are not stored in a controlled environment.

Hormonal receptor expression in EC was also lower compared to the previous studies [40, 41], and this could probably be attributed to weak staining or biological behavior of the EC, which cannot be justified. Low hormonal receptor expression is common in high grade EC [42], but, in this study, tumor grades were equally distributed across all EC cases. Interestingly, we observed only one case (3%) (41-year-old patient with endometrioid carcinoma) that showed abnormal mismatch repair, contrary to 13%, as reported previously [43]. MSH6 was lost, indicating probably Lynch syndrome. This loss suggests that Lynch Syndrome likely exists in East Africa, but more extensive studies on endometrioid carcinomas are needed to assess its prevalence in the East African population.

## Conclusion

Our data suggest an extremely low number of ovaria cancer in selected East African populations compared to the Alberta population. The main limitation was a likely but unknown selection bias. Correct ovarian carcinoma histotyping is vital as some low stage patients could be spared by aggressive chemotherapy. In a resource-limited setting, simple improvement of the basic H&E stain could have a substantial impact on accurate diagnosis according to current WHO criteria. Diagnosis of rare molecularly defined entities requiring access to molecular testing remains a challenge for pathologists in a resource-limited setting and calls for solidarity from resource-rich countries.

## Supplementary information

**Additional file 1: Supplementary table 1.** Immunohistochemical protocols.

**Additional file 2: Supplementary table 2.** Concordance of the correctly classified original diagnosis (Kappa=0.3430, 96% CI: 0.2774-0.4087).

## Data Availability

Submitted as additional files.

## Abbreviations

AGCT: Adult granulosa cell tumor
BMC: Bugando Medical Center
CCC: Clear cell carcinoma
EC: Endometrioid carcinoma
EPH: Epithelia
FFPE: Formalin fixed paraffin embedded
GCT: Germ cell tumors
HGSC: High grade serous carcinoma
IHC: Immunohistochemistry
JGSC: Juvenile granulosa cell tumor
KCMC: Kilimanjaro Christian Medical Center
LGSC: Low grade serous carcinoma
LMPH: Lymphomas
MC: Mucinous carcinoma
NM: Non malignant
SCST: Sex-cord stromal tumors
SCCOHT: Small cell carcinoma of the ovary, hypercalcemic type
TMA: Tissue microarray
WHO: World Health Organization
