# Supplementary material for: Ovarian cancer: diagnostic accuracy and tumor types distribution in East Africa compared to North America
Source: Diagn Pathol. 2020 Jul 16;15:86. doi: 10.1186/s13000-020-01000-3 (PMC7364467; doi:10.1186/s13000-020-01000-3)
Supplement: Supplementary file 1 — Additional file 1: Supplementary table 1. Immunohistochemical protocols. [file 13000_2020_1000_MOESM1_ESM.docx]

**Supplementary table 1. Immunohistochemical protocols**

|  | **Antibody** | **Place** | **Platform** | **Antibody company/clone** | **Dilution** | **Pre-treatment** | **Interpretation** |
| --- | --- | --- | --- | --- | --- | --- | --- |
| **1** | AE1/AE3 | CLS | Dako Omnis | Monoclonal mouse. Dako, cloneAE1/AE3 | Ready-to-use | 30 min HIER | Negative: Absence of staining  Positive: Any staining ≥ 1% of tumor cells |
| 2 | WT-1 | CLS | Dako Omnis | Monoclonal mouse. M. Leica Biosystems, Clone WT41 | 1:40 | 30 min HIEP | Negative: Absence of staining  Positive: Any staining ≥ 1% of tumor cells |
| 3 | p53 | CLS | Dako Omnis | Monoclonal mouse. Dako, Clone DO-7 | Ready-  to-Use | 30 min HIER | Wild type: Heterogeneous nuclear staining between ≥ 1% and < 70%  Abnormal: Complete absence of nuclear staining in presence of internal control or diffuse nuclear staining in ≥ 90% of tumor cells |
| 4 | Napsin-A | CLS | Dako Omnis | Monoclonal mouse. M. Leica Biosystems, Clone IP64 | 1:400 | 30 min HIER | Negative: Absence of staining  Positive:  Positive: Any staining ≥ 1% of tumor cells |
| 5 | PR | CLS | Dako Omnis | Monoclonal mouse. Dako, clone PgR 1294 | 1:50 | 10 min HIER | Negative: Absence of staining  Positive: Any staining ≥ 1% of tumor cells |
| 6 | ER | CLS | Dako Omnis | Monoclonal rabbit. Thermofisher, clone SP1 | 1:50 | 20 min HIER | Negative: Absence of staining  Positive: Any staining ≥ 1% of tumor cells |
| 7 | P16 | CLS | Dako Omnis | Monoclonal mouse. CINtech,clone E6H4 | Ready-  to-Use | 30 min HIER | Normal staining: Patchy/ heterogeneous staining  Abnormal staining: Complete absence of staining or Block staining ≥ 95% of tumor cells |
| 8 | Vimentin | CLS | Dako Omnis | Monoclonal mouse. Dako, clone V9 | Ready-  to-Use | 30 min HIER | Negative: Absence or focal in <50% of tumor cells  Positive: Diffuse staining ≥ 50% of tumor cells |
| 9 | PAX8 | CLS | Dako Omnis | Monoclonal mouse. Cell Marque. Sigma-Aldrich, clone MRQ-50 | Ready-  to-Use | 10 min HIER | Negative: Absence of staining  Positive: Any staining ≥ 1% of tumor cells |
| 10 | Inhibin | CLS | Dako Omnis | Monoclonal mouse. Dako, clone R1 | Ready-  to-Use | 20 min HIER | Negative: Absence of staining  Positive: Any staining ≥ 1% of tumor cells |
| 11 | Melan A | CLS | Dako Omnis | Monoclonal mouse. Dako, clone A103 | Ready-  to-Use | 20 min HIER | Negative: Absence of staining  Positive: Any staining ≥ 1% of tumor cells |
| 12 | OCT-4 | CLS | Dako Omnis | Monoclonal mouse. Cell Marque. Sigma-Aldrich, clone MRQ-10 | Ready-  to-Use | HIER | Negative: Absence of staining  Positive: Any staining ≥ 1% of tumor cells |
| 13 | Glypican 3 | CLS | Dako Omnis | Monoclonal mouse. Cell Marque. Sigma-Aldrich, clone IG12 | Ready-  to-Use | HIER | Negative: Absence of staining  Positive: Any staining ≥ 1% of tumor cells |
| 14 | PMS2 | CLS | Dako Omnis | Monoclonal rabbit. Dako, clone EP51 | 1:40 | 20 min HIER | Loss: Absent nuclear staining in tumor cells with positive staining in stromal and lymphocytes  Retained: Any positive (>10%) nuclear staining in tumor cells |
| 15 | MSH6 | CLS | Dako Omnis | Monoclonal rabbit. Dako, clone EP49 | 1:50 | 20 min HIER | Loss: Absent nuclear staining in tumor cells with positive staining in stromal and lymphocytes  Retained: Any positive (>10%) nuclear staining in tumor cells |
| 16 | FOXL2 | Vancouver | Ventana Benchmark | Polyclonal goat, Imgenex, San Diego, CA | 1:25 | 10 min HIER | Negative: Absence of staining  Positive: Any staining ≥ 1% of tumor cells |
| 17 | TTF3 | APRL | Dako Omnis | Monoclonal mouse. Abnova, Clone 3D9 | 1:50 | 20 min HIER | Negative: Absence or focal in <50% of tumor cells  Positive: Diffuse staining ≥ 50% of tumor cells |
| 18 | ARID1A | APRL | Dako Omnis | Polyclonal rabbit. Sigma-Aldrich, clone | 1:500 | 10 min HIER | Loss: Absent nuclear staining in tumor cells with positive staining in stromal and lymphocytes  Retained: Retained staining in ≥ 10% of tumor cells |
| 19 | SATB2 | APRL | Dako Omnis | Monoclonal rabbit. Abcam clone EPNCIR130A | 1:800 | 30 min HIER | Negative: Absence of staining  Positive: Any staining ≥ 1% of tumor cells |
| 20 | BRG1 | APRL | Dako Omnis | Monoclonal rabbit. Abcam, clone EPNCIR111A | 1:50 | 20 min HIER | Loss: Absent nuclear staining in tumor cells with positive staining in stromal and lymphocytes  Retained: Any positive (>5%) nuclear staining in tumor cells |
