# Supplementary material for: Ovarian cancer: diagnostic accuracy and tumor types distribution in East Africa compared to North America
Source: Diagn Pathol. 2020 Jul 16;15:86. doi: 10.1186/s13000-020-01000-3 (PMC7364467; doi:10.1186/s13000-020-01000-3)
Supplement: Supplementary file 2 — Additional file 2: Supplementary table 2. Concordance of the correctly classified original diagnosis (Kappa=0.3430, 96% CI: 0.2774-0.4087). [file 13000_2020_1000_MOESM2_ESM.docx]

**Supplementary table 2. Concordance of the correctly classified original diagnosis**

**(Kappa=0.3430, 96% CI: 0.2774-0.4087)**

| **Original Diagnosis** | **Revised diagnosis (n %)** | | | | | | | | | | | | | | | | |
| --- | --- | --- | --- | --- | --- | --- | --- | --- | --- | --- | --- | --- | --- | --- | --- | --- | --- |
|  | **Carcinoma NOS** | **CS** | **CCC** | **EC** | **GCT** | **HGSC** | **LGSC** | **LYM** | **MC** | **MET** | **MXD** | **NML** | **OSCCHT** | **SC** | **SCST** | **UND** | **Total** |
| **Carcinoma NOS** | **2**  **(2.4)*** | 0 | 2 (2.4) | 12 (14.3) | 6  (7.1) | 36  (42.9) | 1 (1.2) | 0 | 5  (6.0) | 9  (10.7) | 1  (1.2) | 4  (4.8) | 0 | 1 | 4 (4.8) | 1 (1.2) | 84 |
| **CS** | **0** | **2**  **(100)*** | 0 | 0 | 0 | 0 | 0 | 0 | 0 | 0 | 0 | 0 | 0 | 0 | 0 | 0 | 2 |
| **CCC** | 0 | 0 | **0*** | 2  (66.7) | 1  (33.3) | 0 | 0 | 0 | 0 | 0 | 0 | 0 | 0 | 0 | 0 | 0 | 3 |
| **EC** | 0 | 0 | 0 | **10**  **(58.8)*** | 0 | 5  (29.4) | 0 | 0 | 0 | 0 | 0 | 0 | 0 | 0 | 2  (11.8) | 0 | 17 |
| **GCT** | 0 | 0 | 0 | 0 | **17**  **(81.0)*** | 2  (9.5) | 0 | 0 | 0 | 1  (4.8) | 0 | 0 | 0 | 0 | 1  (4.8) | 0 | 21 |
| **HGSC** | 0 | 0 | 0 | 1  (7.7) | 1  (7.7) | **10**  **(76.9)*** | 0 | 0 | 0 | 0 | 0 | 0 | 0 | 0 | 1  (7.7) | 0 | 13 |
| **LGSC** | 0 | 0 | 0 | 1  (33.3) | 0 | 0 | **1**  **(33.3)*** | 0 | 0 | 1  (33.3) | 0 | 0 | 0 | 0 | 0 | 0 | 3 |
| **Lymphoma** | 0 | 0 | 0 | 0 | 0 | 0 | 0 | **13**  **(92.9)*** | 0 | 0 | 0 | 0 | 0 | 1  (7.1) | 0 | 0 | 14 |
| **Malignant Brenner tumor** | 0 | 0 | 0 | 0 | 0 | 1  (50.0) | 0 | 0 | 0 | 0 | 0 | 0 | 0 | 0 | 1  (50.0) | 0 | 1 |
| **MC** | 0 | 0 | 2  (8.7) | 6  (26.1) | 1  (GCT) | 2  (8.7) | 0 | 0 | **5**  **(21.7)*** | 4  (17.4) | 0 | 2  (8.7) | 0 | 0 | 1  (4.35) | 0 | 23 |
| **Neuroblastoma** | 0 | 0 | 0 | 0 | 0 | 0 | 0 | 0 | **0** | 0 | 0 | 0 | 1 | 0 | 0 | 0 | 1 |
| **SC** | 0 | 0 | 0 | 0 | 0 | 0 | 0 | 0 | **0** | 0 | 0 | 0 | 0 | **2**  **(100)*** | 0 | 0 | 2 |
| **SCST** | 0 | 0 | 0 | 0 | 1  (4.2) | 1  (4.2) | 0 | 0 | **0** | 0 | 0 | 1  (4.2) | 0 | 1  (4.2) | **20**  **(83.3)*** | 0 | 24 |
| **Total** | 2 | 2 | 4 | 32 | 27 | 57 | 2 | 13 | **10** | 15 | 1 | 7 | 1 | 5 | 30 | 1 | 210 |

*****Concordance (%), CS: Carcinosarcoma, CCC: Clear cell carcinoma, EC: Endometrioid carcinoma, GCT: Germ cell tumors, HGSC: High grade serous carcinoma, LGSC: Low grade serous carcinoma, LYMH: Lymphoma, MC: Mucinous carcinoma, MET: Metastatic, MXD: mixed, NML: Non Malignant OSCCHT: Small cell carcinoma of the ovary Hypercalcemic type, SC: Sarcoma,
